# Genetic Modifications in Bacteria for the Degradation of Synthetic Polymers: A Review

**DOI:** 10.3390/ijms25105536

**Published:** 2024-05-19

**Authors:** Diego Martín-González, Carlos de la Fuente Tagarro, Andrea De Lucas, Sergio Bordel, Fernando Santos-Beneit

**Affiliations:** 1 Department of Chemical Engineering and Environmental Technology, School of Industrial Engineering, University of Valladolid, Dr. Mergelina, s/n, 47011 Valladolid, Spain; diego.marting@uva.es (D.M.-G.); andrea.lucas@uva.es (A.D.L.); sergio.bordel@uva.es (S.B.); 2 Institute of Sustainable Processes, Dr. Mergelina s/n, 47011 Valladolid, Spain

**Keywords:** synthetic polymers, plastics, biodegradation, genetic engineering, PET, PETase, cutinase, esterase

## Abstract

Synthetic polymers, commonly known as plastics, are currently present in all aspects of our lives. Although they are useful, they present the problem of what to do with them after their lifespan. There are currently mechanical and chemical methods to treat plastics, but these are methods that, among other disadvantages, can be expensive in terms of energy or produce polluting gases. A more environmentally friendly alternative is recycling, although this practice is not widespread. Based on the practice of the so-called circular economy, many studies are focused on the biodegradation of these polymers by enzymes. Using enzymes is a harmless method that can also generate substances with high added value. Novel and enhanced plastic-degrading enzymes have been obtained by modifying the amino acid sequence of existing ones, especially on their active site, using a wide variety of genetic approaches. Currently, many studies focus on the common aim of achieving strains with greater hydrolytic activity toward a different range of plastic polymers. Although in most cases the depolymerization rate is improved, more research is required to develop effective biodegradation strategies for plastic recycling or upcycling. This review focuses on a compilation and discussion of the most important research outcomes carried out on microbial biotechnology to degrade and recycle plastics.

## 1. Introduction

### 1.1. Definition and Classification of Plastics

Plastics are a type of synthetic polymeric material based on carbon and hydrogen and with high molecular weight [1]. Industrial-scale plastic production began in the 20th Century, and their use has increased over time [2,3]. Plastics can be classified according to different criteria. For example, depending on the composition of their backbone, they can be homochain polymers (when their backbone is made up exclusively of carbon) or heterochain polymers (if there are other elements, like oxygen and nitrogen, present in their backbone) [4]. Depending on the monomers that they are made of, they can be classified as homopolymers if they only have one monomer or copolymers if they have two or more different monomers [1]. Depending on their thermomechanical properties, they can be thermosets if, during their fabrication, covalent bonds are established between polymer chains, giving them high resistance and a shape that cannot be modified thermically; thermoplastics, if no covalent bonds are established between polymer chains so they can be fused and reshaped; or elastomers if they have a high elasticity [5]. Depending on the presence of benzene rings in their backbone, they can be aromatic if they have benzene rings or aliphatic if they do not have benzene rings [6]. Depending on the raw material they are made from, they can be petrochemical (the raw material is petroleum) or biobased (the raw material is biomass), and depending on their degradability by organisms, they can be biodegradable or non-biodegradable.

### 1.2. Advantages and Disadvantages of Plastics

In general, plastics are lightweight, long-lasting, inert and cheap and easy to produce. Their diversity is such that there are plastics with the ideal characteristics for almost any application. Furthermore, the use of mixtures and alloys, as well as additives, can change the properties of the material and adjust them as desired [1]. As plastics are remarkably diverse and can have vastly different properties, their uses are just as varied. They are used to make, among many others, fibers and textiles, toys, packaging, healthcare instruments, such as syringes and implants, and construction materials for insulation, pipes and cable coatings [1]. Even though plastics are generally cheap and easy to produce and have good mechanical properties, the field of engineering requires especially resistant materials for very specific applications. Though they are more expensive, the so-called “engineering plastics” are used for that purpose as they boast a high performance [1]. Other plastics, known as commodity plastics, are found in everyday items.

**Figure 1:**
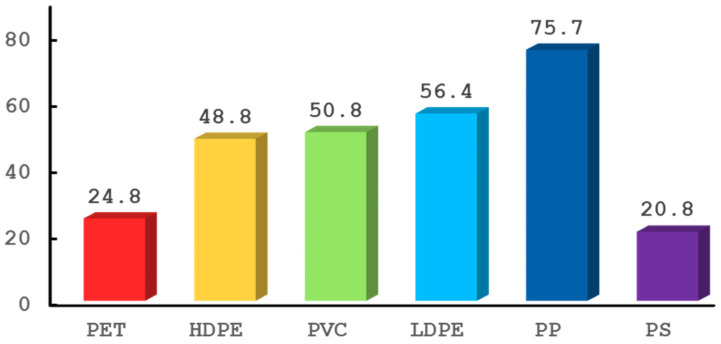
Global production of the 6 commodity plastics (in million metric tons). Data obtained from [10].

The most used commodity plastics are poly(ethylene terephthalate) (PET), high-density polyethylene (HDPE), poly(vinylchloride) (PVC), low-density polyethylene (LDPE), polypropylene (PP) and polystyrene (PS) [2,7,8]. Products made with these plastics can be identified according to the ASTM International Resin Identification Coding System (RIC). The “Others” category includes all other plastics, as well as products made with a mixture of two or more plastics [9]. The global annual production of plastics exceeded 400 million metric tonnes (400 Mt) in 2022, 362.3 of which were new petrochemical plastics. Commodity plastics make up the following percentages (see Figure 1): PET 6.2%, HDPE 12.2%, PVC 12.7%, LDPE 14.1%, PP 18.9% and PS 5.2% [10]. Out of the different uses, most plastic is destined for packaging. In 2019, 142 Mt (31%) of plastics were used in packaging [3,7]. Between 1950 and 2022, more than 10 billion metric tonnes (10.000 Mt) of plastics were produced [2,10,11].

Despite their useful properties, the use of plastics has two main disadvantages, which have to do with the beginning and the end of their lifetime: the first one is the fact that most plastics are petrochemical, and the second one is the amount of non-biodegradable waste generated. Regarding their origin, the reserves of petroleum are limited as petroleum is a non-renewable resource, and given the production rate in 2020, it is estimated that reserves will last for 50 years [12]. For the purposes of this article, the waste generated is the most relevant problem.

## 2. Bioplastics as an Alternative to Petroleum-Based and Non-Biodegradable Plastics

The most recent assessments estimate that out of the 9200 Mt of plastics produced until 2017, 6500 Mt (70%) have become waste. Of this waste, 5000 Mt (54%) ended up in landfills or were released into the environment [13]. This plastic waste has reached virtually every corner of the planet. The five so-called garbage patches are notorious for accumulating 250,000 tons of plastic [7], but plastics are also found elsewhere. They have been found in rivers, lakes, drinking water and table salt and even in extremely remote places, such as the seabed and both polar regions. They have reached such areas mostly in the form of micro- and nanoplastics, whose size allows them to travel much further and even enter organisms’ cells [14]. Due to their composition and structure, plastics can remain in the environment for thousands of years [15], and their effects on living beings’ health, while a complicated matter that is still under study, are undoubtedly severely negative. This has been researched especially in marine ecosystems, where they can cause harm to many different organisms [8,16,17], but they are also apparent in terrestrial ecosystems [18] and human health [19,20]. Moreover, the additives used to modify the properties of plastics can also have toxic effects on organisms [21], and plastics can adsorb and accumulate heavy metals, persistent organic contaminants and even pathogens [22].

Due to their harmful effects on the environment, plastics, like any other type of waste, have to be managed appropriately. Recycling rates are overall very low, with only 600 Mt (6.5%) of plastics produced worldwide until 2017 having been recycled and more than 5000 Mt of them ending up in landfills or being released into the environment [13]. Landfilling plastic waste, though simple, is not an adequate waste management strategy, as landfills can leach microplastics [23,24] as well as other contaminants [25]. Moreover, plastic waste in the environment does not remain permanently unchanged, as it slowly undergoes physicochemical changes that partially break it down. These changes include photodegradation, hydrolysis and thermo-oxidation. However, this transformation results mostly in the fragmentation of plastic rather than its mineralization, generating smaller plastic fragments (micro- and nanoplastics) that still remain in the environment. In order for plastics to be mineralized and, therefore, completely removed from the environment, biological degradation is required [26]. This biological degradation can be performed by different organisms but, even if plastics end up mineralized by the action of these organisms, the degradation rates are so slow and the plastic permanence is so high that most plastics are considered non-biodegradable. Given the problems derived from the properties of the most used plastics, a new type of material has received a lot of attention: bioplastics.

**Figure 2:**
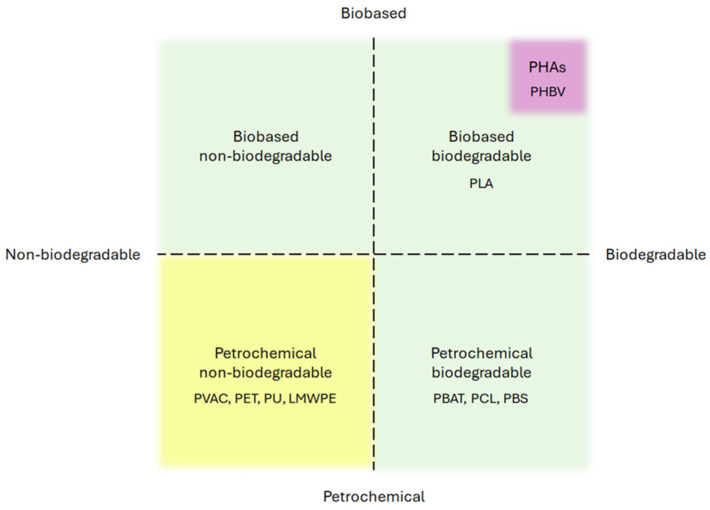
Classification of plastics according to the raw material and biodegradability. PHAs (including a major type, i.e., PHBV) constitute a special category of plastics since they are completely natural.

Bioplastics are a type of plastic that differs from conventional plastic in at least one of two ways: the raw material they are made from or their biodegradability. Bioplastics can either be made from biomass—biobased plastics—or be biodegradable or have both properties [27]. A particular type of bioplastics is poly(hydroxyalkanoates) (PHAs). They are naturally produced by some microorganisms, mostly bacteria, for energy storage [28] and are both biobased (as they are produced by an organism that can be grown with renewable feedstock) [29] and biodegradable (as they can be easily enzymatically degraded) [30]. Just like plastics in general, bioplastics are very varied, so grouping them in the bioplastic category is not particularly informative of their properties, especially without specifying which criteria make them such. Therefore, when discussing individual plastics, this work will specify if they are petrochemical or biobased and biodegradable or non-biodegradable (Figure 2). The main benefits of using bioplastics are summarized in the fact that biobased plastics do not involve the use of petroleum, and biodegradable plastics can be fully mineralized by organisms. However, bioplastic production still has an environmental impact when the whole life cycle is considered [31], and regarding biodegradable plastics, this property does not mean that they can be released into the environment without consequences [32].

Biodegradable plastics can remain in the environment for long periods of time [33,34,35,36] and carry potentially toxic substances [37]. They can even act as a reservoir of microbes with antibiotic-resistance genes [38], so their waste should be managed, too [39]. In the case of biodegradable plastics, the preferred waste management strategy is carrying out their degradation by organisms. There are international standards developed to clearly define the conditions and timescale of biodegradation [40,41,42,43,44,45,46], but their use is not mandatory, so the criteria to determine the biodegradability of plastics can be quite arbitrary. However, plastic is generally considered biodegradable if there are organisms that can mineralize it in a reasonable amount of time in controlled conditions, which must always be specified.

## 3. Biodegradation of Plastic Polymers

The last few decades have seen an increasing interest in both biodegradable plastics and organisms that can degrade plastics. As new species and strains are discovered and genetic engineering is used to improve the enzymes involved in biodegradation, plastics that were previously considered non-biodegradable can now be degraded by one organism or another and to a greater or lesser extent. Therefore, the categories of biodegradable and non-biodegradable plastics are not fixed and can change over time, but in this work, we will use the usual classification.

Plastic-degrading microorganisms include bacteria, fungi and microalgae [47]. This review focuses on bacterial strains. Although genetic engineering approaches have been performed for decades, only a few bacteria have been modified to improve their capability of degrading these synthetic plastics or to grant them this characteristic by modifying their metabolism. More specifically, the modifications performed consist of the heterologous expression of enzymes that come from other organisms, mutating/changing specific amino acids of the enzymes and the addition of domains or other protein structures to the enzymes. Different techniques have been used in order to carry out these modifications, such as metagenomic analysis, directed and non-directed mutagenesis and the use of fusion proteins or chimeras.

This review is a compilation of the studies in which bacteria have been genetically modified, whether to grant them or enhance their ability to degrade plastics or simply to perform genetic modifications and will only mention the polymers for which modified bacteria have been obtained. Table 1 shows the modified bacterial strains that have been developed for the degradation of plastic polymers according to research published in the scientific literature (i.e., PubMed, Scopus, etc.).

Since PET is one of the most produced plastics worldwide and its degradation is challenging, a special focus is put in this review in relation to the microbial biodegradation strategies developed specifically for this polymer (summarized in Table 2).

### 3.1. Biodegradation of PET

**Figure 3:**
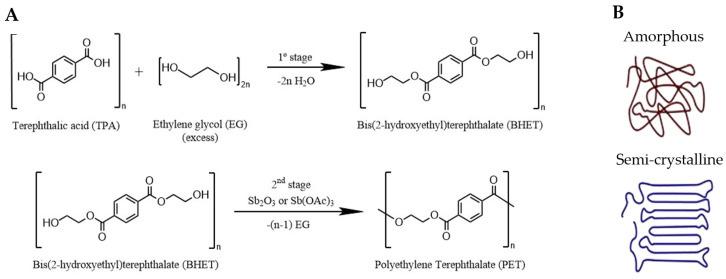
(**A**) PET industrial formation process using BHET as intermediate. Based on [5]. (**B**) Molecular structure of amorphous (**up**) and semi-crystalline (**down**) materials of PET. Adapted from [77].

PET is one of the most produced petrochemical synthetic polymers, accounting for around 6.2% of global plastic production in 2022 [10], and its market size surpasses other highly produced plastics, such as HDPE, PVC or PP [74]. The main reason why PET is so produced is because its molecular structure offers a lot of versatility, making it essential in our daily lives. PET is a long semi-aromatic thermoplastic polyester chain produced from ethylene glycol (EG) and terephthalic acid (TPA). Its production has two steps. First, the union of two EG molecules and one TPA molecule by esterification generates an intermediate molecule, bis(2-hydroxyethyl) terephthalate (BHET). Secondly, BHET along with catalysts, such as Sb_2_O_3_ or Sb(OAc)_3_, is subjected to a process of polymerization, creating the long chain through ester bonds (Figure 3A) [5,75]. When it comes to the manufacturing process, amorphous and semi-crystalline PET are produced depending on the thermal processing undergone during the polymerization. The main difference between these two types lies in the intrinsic viscosity and molecular weight. In the case of the amorphous polymer, the long polyester chains are randomly set out, resulting in a more flexible plastic. On the other hand, semi-crystalline materials are formed by amorphous domains and chains arranged in an orderly way, making the material more resistant and less ductile (Figure 3B). These differences in its molecular structure make it both chemically and thermally stable. This is what makes PET a strong and durable compound, ideal for a wide variety of applications, such as synthetic fibers for the textile industry, water bottles and packaging [5,76].

Despite the advantages provided by PET, it also presents the serious drawback of what to do with it after its useful life. Most PET comes to an end accumulated in landfills. It is estimated that it takes around 300 years to decompose, degrading over time because of solar radiation and heat, among other factors, and releasing harmful chemical compounds to the environment. The estimate increases if its degradation is not accelerated by heat or solar radiation, reaching 2500 years or more. PET is so difficult to degrade due to its physicochemical properties, which make it resistant to decomposition by water and organic and inorganic compounds [73,76].

For this reason, there are a series of methods, which involve mechanical, chemical and biological methods, to recycle and reuse this polymer, producing fibers and fabrics. The mechanical methods are the most widespread, but they are also very expensive, while in the case of chemical methods, the process can be harmful to the environment [70,78].

In contrast with the previously mentioned methods, biological methods are on the rise because they do not damage the environment. These methods are still in development and are based on the use of cells as factories of enzymes able to break the bonds of PET, releasing the monomers of which it is composed. After that, those different monomers can be subjected to a valorization process to generate high value-added molecules, such as polyhydroxyalkanoates, vanillic acid, gallic acid, lycopene, glycolic acid, pyrogallol, catechol and muconic acid, which can be used as flavors, cosmetics, sanitizers and animal feed and in pharmacy, among other uses [78,79].

#### 3.1.1. Enzymes Involved in the PET Degradation Pathway

The reason why PET can be degraded by enzymes is because a lot of them are unspecific regarding their substrates. The current literature offers many names given to these enzymes, such as PET hydrolases, PET esterases, PET cutinases, PET depolymerases or PETases. PET esterases, PET cutinases and PET depolymerases are hydrolytic enzymes that are able to break the ester bonds of biological molecules, like suberin and cutin, and due to their unspecificity, it turns out that they can break those of PET as well. On the other hand, PETases are hydrolytic enzymes more specific to PET [80,81]. Due to the specificity, there are small differences between the different types of enzymes, such as PET cutinases, which can hydrolyze the ester bonds of aliphatic and aromatic molecules, while PETases can only hydrolyze bonds of aromatic molecules [54,65,69]. To simplify the following explanation, we will refer to all of them as PETases unless it is otherwise stated, with the understanding that PETases are enzymes that hydrolyze PET.

**Figure 4:**
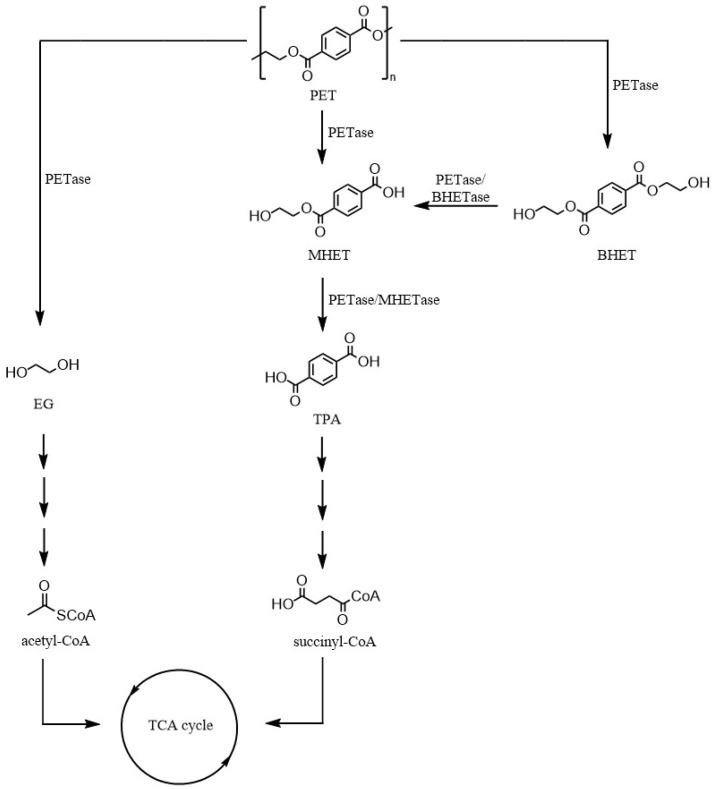
Chemical structures of PET and products related to its hydrolyzation. Each arrow represents an enzymatic reaction. The presence of multiple arrows, like between EG—acetyl-CoA and TPA—succinyl-CoA, refers to the fact that there is more than one chemical reaction between one molecule and another. Adapted from [80].

The hydrolyzation pathway of PET is summarized in Figure 4. In most cases, PETases are capable by themselves of firstly depolymerizing the PET chain and secondly degrading its monomers—mono(2-hydroxyethyl) terephthalate (MHET) and BHET—to finally form TPA and ethylene glycol. PETases can directly produce MHET and TPA, or In contrast, if BHET is generated, they can degrade it to MHET [5,78]. However, there are some PETases that are not able to carry out both steps by themselves, as is the case with PE-H (*Pseudomonas aestusnigri*) and *Is*PETase (*Ideonella sakaiensis*). Both can only depolymerize PET because they cannot break the ester bond of MHET, which accumulates [82]. In contrast, there are enzymes capable of degrading only MHET (MHETases). *Is*MHETase is an example, being able to degrade MHET to finally produce ethylene glycol and TPA. Although there are enzymes that do not carry out both degradation actions by themselves, they can complement each other. *Is*MHETase and *Is*PETase were found in *I. sakaiensis* 201-F6 when this strain was discovered in a medium whose only carbon source was PET [81]. Recently, a new category of enzymes has been discovered, BHETases, which specifically catalyze the transition from BHET to MHET. Two BHETases have been characterized, *Chry*BHETase from *Chryseobacterium* sp. and *Bs*Est from *Bacillus subtilis* [83].

As a consequence of the cooperation between PETases and MHETases or the action of only PETases, PET is hydrolyzed, and TPA and ethylene glycol are released, and they can be metabolized (Figure 4). TPA presents a biochemical pathway to finally be degraded to succinyl-CoA. Regarding ethylene glycol, it can be metabolized to obtain acetyl-CoA [78]. Both succinyl-CoA and acetyl-CoA are incorporated into the tricarboxylic acid cycle (TCA) to obtain energy for the bacteria and, in certain cases, some products of interest. For example, species *I. sakaiensis* and *Geobacter sulfurreducens* when cocultivated can degrade PET to ethylene glycol and generate electricity [84]. In another study, a *Rhodococcus josii* strain PET metabolized the PET hydrolysate and synthesized lycopene, which is used in medicine for cancer treatments [85]. One example of a modified bacteria is *Escherichia coli* modified to degrade PET and produce vanillin, present in cosmetic and food industries [70]. The cleavage of the ester bond happens inside the enzyme’s active site, and the ability of different enzymes to hydrolyze PET comes as a consequence of a nucleophilic similarity produced by three amino acids conserved in PETases, Ser-His-Asp. In contrast, the adjacent sequences to these amino acids are different among enzymes, varying their selectivity to substrates [50,63].

There are two proposed classifications for PETases in the literature. One of them is based on their sequence, while the other one is based on their protein structure. According to the sequence-based classification, PETases can be classified as type I and type II enzymes [5]. Type I enzymes have one C-terminal disulfide bridge, and type II enzymes have two, with one of them being close to the active site. This additional disulfide bridge provides type II enzymes more thermal stability and plays a key role in the hydrolytic action [5,63,86]. Another characteristic that differentiates both types is the amino acids present in the active site. Type I enzymes, such as LCC and Cut190, which will be mentioned later, have His159 and Phe/Tyr238 residues, and type II enzymes present Trp and Ser in the same locations. In addition, type II enzymes are subdivided into type IIa if they have Phe or Tyr residues instead of Ser238 or type IIb if they maintain Ser, as is the case with *Is*PETase. As an exception, type I enzymes may not have the disulfide bridge. This is the case with PET27 and PET30 from *Aequorivita* sp. CIP111184 and *Kaistella jeonii*, respectively. According to the structure-based classification, PETases can have three different structures, with *Is*PETase, *Fusarium oxysporum* cutinase and *Chloroflexus* sp. MS-G cutinase representing Structures 1, 2 and 3, respectively [87]. For example, *Is*PETase presents an α/β-hydrolase fold and a core made up of eight β-strands and six α-helices, as well as a highly polarized surface [65].

#### 3.1.2. Modifications of Bacteria and Enzymes to Improve PET Degradation

Since the discovery of PET-degrading enzymes in the last century, efforts have been made to improve their degradation rate, either by searching for new enzymes and heterologously expressing them in other microorganisms or by modifying the existing ones. It was not until 2016 that the first PETase (*Is*PETase) was discovered in *I. sakaiensis* 201-F6, a specific enzyme that allowed the bacteria to use PET as its major carbon and energy source. The experiments were carried out at 30 °C and pH 7, and this enzyme was compared with three other enzymes with hydrolytic activity toward PET [81]. *Is*PETase has an α/β hydrolase fold like cutinases but with a larger active site [65]. After this discovery, modifications of this *Is*PETase have been carried out in order to obtain a higher degradation rate. The importance of the amino acids of the active site or close to it is emphasized, as will be seen afterward with cutinases. In addition, the presence of one amino acid or another may affect the enzyme–substrate interaction affinity and, therefore, the degradation of the polymer [86].

However, before *Is*PETase was discovered, different genetic engineering methods had already been carried out to increase the degradation speed of esterases and cutinases [55]. Site-directed mutagenesis was used to specifically modify the sequence close to the active site of a cutinase from *Fusarium solani pisi*. Mutations were carried out to create a bigger space in the active site to facilitate the entrance of the polymeric chain. The modifications that created a more hydrophobic binding site resulted in the greatest PET degradation compared with the native enzyme. PET was degraded at a rate of 12 mg/h/mg of modified cutinase at pH 8 and 50 °C, while the native enzyme degraded 0.05 mg/h/mg of the enzyme at pH 7.0 and 55 °C.

Continuing with the idea of increasing hydrophobicity, in 2011, two amino acids were removed from the active site of the cutinase Tfu_0883 from *Thermobifida fusca*, expanding the space and improving its capacity to hydrolyze polyesters due to better PET adsorption. In the same experimental conditions, after 4 h, the native enzyme reached a spectral value (*k*/*s*) of 0.1, while the modified enzyme reached almost 0.2. Stretching the time, at 48 h, the values were around 0.15 and 0.3, respectively [56]. In addition, the amino acids of surface regions outside the active site turned out to have a direct action in PET hydrolysis, as mentioned in Herrero-Acero et al. [57]. In this study, the cutinase Thc_Cut2 from *Thermobifida cellulosilytica DSM44535* was modified by exchanging amino acids in the surface region. It was shown that exchanging a surface positive residue, like arginine, for a non-charged one, like asparagine and serine, makes a modified enzyme with a higher affinity for PET, according to their *k_cat_*/*K_m_* values. In this case, the modifications produced a variation in the hydrolytic capacity of the enzymes, but the hydrophobicity did not change at any time. It is speculated that these changes in the amino acid sequence may decrease the size of the active site, stabilizing the region. 

A similar idea is mentioned in Kawai et al. [58], where CUT190 cutinase from *Saccharomonospora viridis* AHK190 was triple-mutated to Ser226Pro/Arg228Ser/Thr262Lys. It was previously known that in its native state, it is able to degrade different polymers, such as PCL, PBSA, PBS and PHB, among others. However, these modifications resulted in an enzyme with more activity and higher thermostability. A possible explanation for this result was that a polar amino acid with a positive charge, such as arginine, close to the binding site, could interact with negative charges that may be present in the substrate molecules [61]. For this reason, arginine was replaced by serine. In addition, it was concluded that the addition of Ca^2+^ to the medium provides greater thermostability to CUT190 [58]. In another study, Han et al. [61] obtained an enzyme with a mutation near the substrate binding site that facilitated the union with the substrate and increased its thermostability to 5 °C higher than the native enzyme.

In contrast, Austin et al. [65] achieved a modified *Is*PETase exchanging two amino acids—Ser238Phe/Trp159His—conserved in cutinases next to the active site. The modified enzyme had a smaller binding site and resulted in a slightly higher degradation rate of PET and polyethylene-2,5-furandicarboxylate (PEF), a semiaromatic polyester derived from PET. Hydrophobicity is mentioned to be relevant in the adsorption of the polymer by the active site. In the following work with *Is*PETase, eight modified enzymes were obtained. Three of them with modifications in their active site—Arg61Ala, Leu88Phe and Ile179Phe—resulted, respectively, in higher PET degradation rates of 13.5 mg PET/µmol PETase·L^−1^ per day, 17.5 mg PET/µmol PETase·L^−1^ per day and 22.5 mg PET/µmol PETase·L^−1^ per day compared with the native *Is*PETase of 8.2 mg PET/µmol PETase·L^−1^ per day. In two of them, the hydrophobicity remained constant, and in the third, it increased. However, the other five modified enzymes obtained lower results than the native one. In three of these, the hydrophobicity increased, and only in one mutant, it decreased [66]. Therefore, according to these studies, not only the hydrophobicity in the active site can influence PET depolymerization, but the type of amino acid that is modified can also affect it.

Focusing on another parameter, in 2019, it was tested to see if a more thermostable *Is*PETase was able to degrade PET at a faster rate. Native *Is*PETase has a Tm of 48.81 °C, while the obtained triple-mutated *Is*PETase—Ser121Glu/Asp186His/Arg280Ala—had a Tm of 57.62 °C. This mutant released 14 times more monomers at 40 °C than the native enzyme, obtaining an enzymatic activity of 120.9 µM and 8.7 µM, respectively [67].

In addition, there are genetic engineering works based on metagenomics, such as in Sulaiman et al. [54], where they cloned a leaf-branch compost cutinase (LC-cutinase or LCC), whose highest identity was with a *Thermomonospora curvata* lipase with 59.7%, and degradation of PET was achieved under the conditions of 50 °C and pH 8 at a rate of 12 mg/h/mg of the enzyme. This result is much greater when compared to *T. fusca* cutinase at 55 °C and pH 7, which consumes 0.05 mg of PET/h/mg of the enzyme [88].

The main drawback of expressing LC-cutinase is that it forms aggregates with itself due to electrostatic interactions as a result of increasing the temperature. This produces an inhibition in the depolymerization of PET. For this reason, it was tested to see if glycosylating LC-cutinase could prevent this. The result was that glycosylated LC-cutinase began to form aggregates at 10 °C above its native protein [62].

Following the idea that glycosylation can influence enzymes’ properties, another study used the previously mentioned cutinase Thc_Cut1 from *Thermobifida cellulosilytica* and generated two mutated cutinases without glycosylation sites. All were able to hydrolyze polyesters, such as PET, poly(butylene succinate) (PBS) and poly(3-hydroxybutyrate-*co*-3-hydroxyvalerate) (PHBV), but one of the mutants had higher PBS-degrading activity than the native enzyme [51]. Metagenomics was also used by Wei et al. [59], exchanging amino acids from the structure of a cutinase obtained with this technique for others from TfCut2, a polyester hydrolase from *Thermobifida fusca KW3*. The best mutants were those with modifications near the binding site. The mutations Gly62Ala and G62Ala/I213Ser achieved a weight loss of 42% of PET films, almost three times more compared to the native protein. This may be due to the fact that the wild-type enzyme is inhibited by MHET, and the substitution of these amino acids may decrease the affinity of the mutants for this molecule. To avoid the problem of inhibition by MHET, Barth et al. [60] used the dual-protein system with TfCut2 and the carboxylesterase TfCa, both from *T. fusca* KW3. With this, TfCut2 degraded PET, and TfCa achieved the same with BHET and MHET. This dual system generated 2.4 times more TPA than using TfCut2 alone. On the other hand, Tournier et al. [69] obtained a modified LC-cutinase with a higher affinity for PET and higher thermostability. This was achieved by exchanging two amino acids, previously tested using in silico methods, with which the new enzyme presented a disulfide bridge. This bridge conferred greater strength in the bond to the substrate, and the other two modifications improved thermostability. In its native state, this enzyme depolymerized 50% of the amorphous PET, while the modified LC-cutinase reached 90% after 10 h. Regarding productivity, it produced 16.7 g of TPA per liter per hour as a consequence of degrading PET. The optimal ratio to obtain these results was 3 mg of mutated enzyme/per g of PET. 

Another article based on metagenomics modified the anaerobic bacteria *Clostridium thermocellum* to constitutively express and secrete LC-cutinase in large quantities. After 14 days incubating anaerobically at 60 °C, more than 60% of amorphous PET was degraded. It was 50 mg in total, corresponding to more than 2.2 mg each day [68]. 

One interesting aspect is obtaining high-value by-products through the degradation of PET, as shown by Sadler et al. [70]. Following this idea, they created the strain *E. coli* MG1655 RARE. This genetically modified strain is able to degrade PET, release TPA and obtain vanillin. This was made using PETases from *I. sakaiensis* and a de novo pathway to convert TPA to vanillin. To optimize the process, parameters such as temperature and cell permeabilization were improved, and 79% vanillin production was obtained compared to TPA.

In recent years, there has been a trend to create fusion proteins to depolymerize PET. This is the case with Zhu et al. [71]. They modified *E. coli* with the BIND-PETase dimer. It is a dimer in which the so-called biofilm-integrated nanofiber display (BIND) binds to the curli of bacteria. Curli are fibers on the outside of the cell that are involved in forming biofilms. Through this method, PETase bound to BIND remains on the surface of the cell and degrades the polymer. It is a system that provides thermostability to PETase and makes it able to degrade 9.1% semi-crystalline PET and microplastics between 2.5 and 50 mg.

Based on the protein fusion strategy, Chen et al. [72] synthesized two modified LC-cutinases. They based them on the modified LC-cutinase created by Tournier et al. [69] called LCC^ICCG^ and previously mentioned, which had higher catalytic activity and thermal stability but little affinity for PET. These two fusion proteins, LCC^ICCG^-TrCBM and CfCBM-LCC^ICCG^, were made by adding a substrate-binding domain to LCC^ICCG^ from *Trichoderma reesei* and *Cellulomonas fimi*, respectively. These domains were selected in silico based on the principles that domains from thermophilic organisms, domains that bind to ordered crystalline substrates and domains that are smaller than the rest of the protein are more adequate. It was estimated with the experimental results that the affinity for the polymer increased 1.4 fold in LCC^ICCG^-TrCBM and 1.3 fold in CfCBM-LCC^ICCG^, while they degraded 3.7% and 24.2% more PET, respectively. 

Another recent project using this technique appears in Li et al. [73]. The researchers modified the non-pathogenic, moderate halophile *Vibrio natriegens* with a chimera of *Is*PETase and *Is*MHETase from *I. sakaiensis*, with the aim of degrading PET present in seawater. PET was almost completely transformed to TPA, and it was determined that the strain would take 24 years to completely decompose 1 g/L of PET. The creation of chimeras is a novel technique that still has a long way to go in relation to the degradation of synthetic polymers.

Concluding with this polymer, numerous techniques for its depolymerization have been carried out to improve the efficiency of enzymes or modify bacteria with these PETases. Today, research is still needed to obtain more efficient methods for bacteria. This is due to the physical–chemical structure of PET and its own disposition in space, with the semi-crystalline structure being more recalcitrant. The use of enzymes to treat the problem of this polymer has the advantage of being harmless to living beings and the environment, so further research should be carried out.

### 3.2. Biodegradation of Other Plastic Polymers

Genetic engineering approaches carried out to degrade synthetic polymers have not only focused on PET but also on other polymers, as described below.

#### 3.2.1. Polyethylene (PE)

PE is a particular type of plastic that results from the polymerization of ethylene. A lot of plastics in this group are copolymers of ethylene and olefins, the latter of which result in branches in the plastic’s structure. This results in a wide variety of polyethylenes with different properties [89]. Multiple organisms have been discovered that can degrade different types of polyethylene [90,91,92].

In a study of genetic engineering, Gyung Yoon et al., who expressed the alkane hydroxylase gene (alkB) of *Pseudomonas* sp. E4 in *E. coli* BL21, found that it was able to degrade low-molecular-weight polyethylene (LMWPE) [53]. Kong et al. bound the latex clearing protein derived from *Streptomyces* sp. strain K30 (LcpK30) to the anchor peptide LCI and expressed it in *E. coli* in order to improve its ability to degrade low-density polyethylene (LDPE) [93].

#### 3.2.2. Poly(butylene Adipate-*co*-terephthalate) (PBAT)

PBAT is a long-chain aliphatic–aromatic copolyester formed by subunits of TPA, adipic acid and 1,4-butanediol linked by ester bonds. It is estimated that its global production was more than 360 million tons in 2018 [94]. Its importance relies on the fact that, like PET, it is a plastic used in daily life due to the advantages it presents. It is easy and cheap to produce, resistant and very versatile due to its chemical structure, which gives it the property of being rigid and flexible [95]. PBAT is produced by the condensation of TPA, 1,4-butanediol and adipic acid, and catalysts with tin, zinc and titanium are used [96]. It is widely used in the textile industry, agriculture and packaging. It is also present in organic waste bags because its ester bonds are more easily degradable by enzymes. Its biodegradability has been demonstrated in previous studies, so it is considered a bioplastic [97]. 

However, its biodegradation rate is low [95]. For this reason, some studies have been carried out with cutinases to better understand the hydrolysis mechanism of PBAT. Due to the presence of ester bonds in PBAT, it is possible to extrapolate the cutinases research carried out on PET to biodegrade PBAT. This happens with Yang et al. [98], where researchers studied the structure of *T. fusca* cutinase TfCut. This enzyme is known to degrade PBAT into its monomers. A double-mutated cutinase, TfCut-DM, was created. TfCut-DM depolymerized all the PBAT into TPA after 48 h of culture, while, at that time, the native enzyme released more 1,4-butanediol-TPA than TPA. On the other hand, genes related to anaerobic living beings, such as *Clostridium botulinum*, have been researched. This is the case with the esterases Cbotu_EstA and Cbotu_EstB, which are expressed heterologously in *E. coli* BL21-Gold(DE3). They have been shown to be able to hydrolyze PBAT. However, some differences in the amino acids of its active site make Cbotu_EstA more efficient than Cbotu_EstB. In silico representations show that the active site of the first protein is larger, which can facilitate the entry of the polymer [49].

#### 3.2.3. Poly(butylene succinate) (PBS)

PBS is an aliphatic polyester formed by the condensation of succinic acid and 1,4-butanediol. In the past, it was produced from compounds derived from petroleum. Nowadays, it is produced from renewable resources, such as sugar cane and corn, through bacterial fermentation [99]. This is a point in its favor in terms of being produced in greater quantities, along with the physical and chemical properties it has. It has a crystalline structure that gives it rigidity and some ductility and a wide temperature range with which to work. Among other uses, it is present in the textile industry and is a possible substitute for polypropylene [100].

The same proteins that hydrolyze PET and PBAT break the ester bonds of PBS. A study on the genetic engineering of cutinases is the one of Gamerith et al. [51], mentioned above for PET. In the article, they used Thc_Cut1 cutinase and two mutants, of which one of them resulted in hydrolyzing the PBS at a higher rate, degrading 92% of the dry weight after 96 h. This confirms its potential for PBS biodegradation.

#### 3.2.4. Polylactic Acid (PLA)

PLA is an aliphatic polyester formed by monomers of lactic acid. In its manufacturing, it is normally made from hydroxyl acids. It is based on bacterial fermentation, as in the case of PBS, to synthesize lactic acid. Later, lactic acid is purified, and the monomers are condensated and linked by ester bonds. PLA is considered a bioplastic that can be chemically depolymerized to lactic acid in recycling processes. PLA is rigid and transparent, and compared to other bioplastics, it has a long durability. Among its uses, it is used as food packaging, for 3D printing, in medicine and the textile industry. PLA can be degraded by cutinases and esterases, as in the case of PET, PBAT and PBS [101]. 

There are almost no genetic engineering studies focused on PLA, partly because the same enzymes can hydrolyze multiple polymers and partly because PLA is not as recalcitrant as others, such as PET, making PLA less interesting when it comes to developing biodegradation strategies. There is, however, a patent on a protease from *Thermus* sp. Rt41A that has been mutated and whose variants are meant to be used to biologically degrade PLA and recover lactic acid monomers [48].

#### 3.2.5. Poly(3-hydroxybutyrate-*co*-3-hydroxyvalerate) (PHBV)

PHBV, one of the most known PHAs, is a copolymer of hydroxyl-butyrate (HB) and hydroxyl-valerate (HV). When compared to PHB, another well-studied PHA, PHBV has better flexibility and a lower melting temperature [102]. The ratio between both monomers determines the plastic’s properties, with a higher content of HB resulting in higher strength and a higher content of HV resulting in higher flexibility and toughness [103]. PHBV’s properties can be improved in different ways [104], for example, by mixing it with agro-residues, which results in green composites that have enhanced physical properties [102], or by forming small crystal nuclei and then drawing the material, resulting in fibers with high tensile strength [105]. PHBV has many different uses [106]. It can be used for coatings and packaging [107,108,109] as well as 3D printing [110]. PHBV can also be used in the textile industry, especially when blended with poly(lactic acid) (PLA), a biobased and biodegradable plastic [111], and in cosmetics as microbeads for exfoliants [112]. The medical field has also found various uses for PHBV. For example, it can be used to make biocompatible scaffolds [113,114] or slowly release drugs like insulin [115] or drugs for treating tumors [116]. Each of the monomers of PHBV is synthesized in a different metabolic pathway. In one pathway, acetyl-CoA is transformed into acetoacetyl-CoA, which, in turn, is transformed into 3-hydroxybutyryl-CoA. In the other pathway, oxaloacetate is sequentially transformed into threonine, 2-ketobutyrate, propionyl-CoA, 3-ketovaleryl-CoA and R-3-Hydroxyvaleryl-CoA [117].

PHBV waste can be recycled. For example, PHBV-based face masks can be chemically recycled through hydrolysis [118]. However, as PHBV is a biodegradable plastic, carrying out its biodegradation is preferable. Its biodegradability when released to the environment is slow and depends greatly on environmental conditions [119], but PHBV has been estimated to biodegrade in rivers [120], and the highest degradation is reached when in activated sludge [121] or compost [122,123]. PHBV-degrading bacteria have been isolated from soil, specifically *Actinomadura* sp. AF-555 [124] and *Streptomyces* sp. MG [125], but very little is known about specific enzymes that can degrade PHBV. In an aforementioned study, the enzymes Thc_Cut1 and Thc_Cut1_koST from *Thermobifida cellulosilytica* DSM44535, expressed in *P. pastoris*, were able to degrade PHBV, though to a very limited extent [51].

#### 3.2.6. Polyvinyl Acetate (PVAC)

PVAC, also known as PVAc, is a petrochemical plastic that is mostly used as an adhesive, particularly for paper and wood [126], but it has also found a use as part of graphene-derived composites [127]. The monomer, vinyl acetate monomer (VAM), is synthesized through the oxidative addition of acetic acid to ethylene, and acetic acid can be, in turn, synthesized from ethylene [128]. Polymerization reactions in PVAC include emulsion-, suspension- and solution polymerization [129].

It has been reported that fungi can grow on items made from PVAC, suggesting its biodegradation [130], but PVAC is generally considered to be non-biodegradable [128]. Liu et al. [50] developed a chimeric lipase–cutinase (Lip-Cut), originally *from Thermomyces lanuginosus* and *Thielavia terrestris* NRRL 8126, respectively, and expressed it in *P. pastoris.* They studied the use of this Lip-Cut in the process of deinking waste paper and found that the synergy between both enzymes improves the ability of the cutinase to both degrade PVAC and deink paper. Also, regarding paper waste treatment, Liu et al. [131] fused the cutinase from *Humicola insolens* to the *E. coli* anchor peptide OMP25, which can be adsorbed on the polyester polymethylmethacrylate (PMMA). They expressed the fusion protein in *P. pastoris* and concluded that the anchor peptide enhances the cutinase’s ability to degrade PVAC and the so-called stickies in waste paper recycling.

#### 3.2.7. Poly(ε-caprolactone) (PCL)

PCL is a plastic well known for its use in the biomedical field [132,133], as it is biocompatible [134]. It can be used in drug delivery or wound dressing [135], but it stands out as a scaffold for tissue engineering, whether on its own [136] or as part of a blend or composite material [137]. Its monomer is ɛ-caprolactone, and its polymerization can be carried out by opening the monomer’s ring followed by the addition or, alternatively, by polymerizing 6-hydroxycaproic acid [138].

PCL is a biodegradable plastic [139,140], and its degradation is especially high when in anaerobic sludge [141] and compost [142] or when performed by bacteria isolated from compost [143]. Regarding engineered bacteria to degrade PCL, two examples have already been mentioned. Sulaiman et al. [54] found that LC-cutinase could degrade PCL in addition to, and better than, PET. The aforementioned CUT190 cutinase from *Saccharomonospora viridis* AHK190, when double-mutated and expressed in *E. coli* Rosetta-gami B (DE3), had an improved degradation activity of PCL, among other polymers [58].

#### 3.2.8. Polyurethane (PU or PUR)

PU is a polymer synthesized by the union of a diol and a diisocyanate molecule linked by amide bonds. To accelerate the process, catalysts, such as 1,4-diazabicyclo[2.2.2]octane or dibutyltin dilaurate, can be added. Polyurethane is the eighth most consumed polymer in the world, with 18.6 MMt/yr. Its structure can be highly varied, and it has a lot of uses. Its main use is as a foaming agent, but it is also manufactured in thermal insulation processes and to protect against abrasion and corrosion, among other uses. Its drawback is that this manufacturing requires toxic substrates, making it difficult to recycle. For this reason, alternative routes to synthesize it are being sought. In addition, using biodegradation to eliminate this polymer in a harmless way is being studied [144]. PU has amide bonds that are hydrolyzed by amidases, releasing the monomers. To increase the degradation of PU, some researchers improved the adsorption of an amidase from *Nocardia farcinica* to PU by fusing it to a polymer-binding module originally from a polyhydroxyalkanoate depolymerase obtained from *Alcaligenes faecalis* [52]. However, there are not many studies based on the biodegradability of PU.

## 4. Conclusions

The magnitude of plastic usage and the later production of its waste, which in most cases remains in nature for decades, makes the search for alternative bioplastic materials or other compounds a matter of major importance for today’s society. In addition, the biodegradation of these materials and, where possible, the use of their constitutive monomers as carbon and energy sources for the growth of microorganisms that can produce valuable compounds are important goals to achieve [145].

Despite the fact that there are a large number of studies based on genetic modifications, most of them were carried out through modifications in the amino acid sequence. New strategies have been recently tested, such as the use of chimeras or fusion proteins, among others, but these approaches are a minority. Further research in the use of genetic techniques, as well as combining different strategies, is necessary to obtain new strains or proteins with a greater degradative capacity of these synthetic polymers.

An example of a new research field is the use of transcriptional regulators, such as Garrido et al. [146] have carried out. This work tested if the FarA transcription factor in *Aspergillus oryzae* was directly related to the synthesis of CutL1 and HsbA, a cutinase and a hydrophobic surface binding protein that participate in the degradation of poly(butylene succinate-*co*-adipate) (PBSA), a biodegradable polymer. The deletion of FarA produced a mutant with minimal concentrations of *cutL1* and *hsbA* compared with its native strain and without the ability to degrade PBSA. This study differs from the ones described in the main text, as it consists of the basic research of an organism and its genetic regulation. This does not mean that the other studies ignore the subject of gene expression. In contrast, modifying bacteria to express an enzyme requires careful consideration of the genetic elements necessary for its successful production. The difference relies on studying genetic expression in the native organism, which cannot only help us understand their genetic regulation and metabolism but also give us hints on different approaches to enhance plastic degradation and how to use the native organism instead of relying on a different host organism.

It is also relevant to point out that the research tends to focus on enhancing an enzyme’s ability to degrade specific polymers, with almost no studies evaluating how the modifications performed may change specificity toward other polymers. Limiting the analysis to just a few substrates—for which the enzyme is already specific—is comprehensible, but using a wider variety of polymers could indicate if the modifications have a greater effect than expected and may be more useful.

In addition, there is little work on the tridimensional structure of the different enzymes—native or modified—that have been mentioned. Deeper research is necessary on this matter in order to better understand why changes happen regarding the affinity for the substrate or the stability, among other characteristics, according to the exchanged residues. Obtaining the structure of enzymes can be achieved not only through X-ray crystallography but also in silico by modeling. This is especially useful when proteins cannot be successfully purified and crystallized, but modeling goes even further. From molecular dynamics to substrate docking, these tools are used in some of the referenced studies to analyze enzymes’ properties and enhance their activities.

Similar to plastics themselves, approaches to studying their biodegradation are diverse, and this variety often comes with a lack of consistency between studies. The conditions in which the enzymes are used can differ, and some studies do not calculate enzymatic activity and limit themselves to detecting solid plastic disintegration or monomer liberation, and the works that calculate enzymatic activity can use different units. This can sometimes make it complicated to compare their conclusions.

Overall, more research is required to develop effective, i.e., quicker, safer and more efficient, biodegradation strategies for plastics, if possible. This applies not just to the plastics that are briefly addressed in this work but also to all plastics in general. Microbial biotechnology and genetic engineering approaches, together with the current development of artificial intelligence tools that provide a new direction in the study and design of novel of enzymes, can facilitate the generation and optimization of several types of plastic-degrading enzymes and valorization processes.

Nowadays, we are getting closer to achieving this aim thanks to the latest advances in DNA sequencing, metagenomics, bioinformatics, genome mining and machine learning tools, in conjunction with new genetic engineering techniques, such as CRISPR-Cas technologies.

## Figures and Tables

**Table 1:** Heterologous expression of enzymes to degrade PET, PLA, PBAT, PVAC, PCL, PBS, PHV, PU and LMWPE.

Host	Plastic	Enzyme	Origin Species	Ref.
*E. coli* BL21 (DE3)	PLA	Protease (Plasmid: pET26b(+))	*Thermus* sp. Rt41A	[48]
*E. coli* BL21-Gold (DE3)	PBAT	Esterases Cbotu_EstA and Cbotu_EstB	*Clostridium botulinum*	[49]
*E. coli* DH5α	PVAC, PCL	Cutinase (Cut) and lipase (Lip) (Plasmid: pPICZαA)	*Thermomyces lanuginosus* (Lip); *Thielavia terrestris* NRRL 8126 (Cut)	[50]
*E. coli* XL-10	PET, PBS, PHBV	Cutinase 1 (Thc_Cut1) (Plasmid: pMK-T; pPICZαB)	*Thermofida cellulosilytica*	[51]
*E. coli* XL10-Gold	PU	Polyamidase (PA) (Plasmid: pET26b(+))	*Nocardia farcinica* IMA 10152A (PA)	[52]
*E. coli* BL21 Gold (DE3)	PU	Polyamidase (PA) (Plasmid: pET26b(+))	*Nocardia farcinica* IMA 10152A (PA)	[52]
*E. coli* BL21	LMWPE	Alkane hydroxylase (Plasmid: pUC19)	*Pseudomonas* sp. E4	[53]
*E. coli* BL21-CodonPlus (DE3)	PET, PCL	Cutinase	Metagenomical library	[54]

**Table 2:** Heterologous expression of enzymes to degrade PET.

Host	Plastic	Enzyme	Origin Species	Ref.
*E. coli* BL21 (DE3)	PET	Cutinase (Plasmid: pET25b(+))	*Fusarium solani pisi*	[55]
*E. coli* BL21 (DE3)	PET	Cutinase Tfu_0883 (Plasmid: pET20b)	*Thermobifida fusca*	[56]
*E. coli* BL21-Gold (DE3)	PET	Cutinase Thc_Cut2 (Plasmid: pET26b(+))	*Thermobifida cellulosilytica* DSM44535	[57]
*E. coli* DH5α	PET	Cutinase-type polyesterase (Cut190) (Plasmid: pGEM-T; pQE80L)	*Saccharomonospora viridis* AHK190	[58]
*E. coli* Rosetta-gami B (DE3)	PET	Cutinase-type polyesterase (Cut190) (Plasmid: pGEM-T; pQE80L)	*Saccharomonospora viridis* AHK190	[58]
*E. coli* BL21 (DE3)	PET	Cutinase TfCut2	*Thermobifida fusca* KW3	[59]
*E. coli* BL21 (DE3)	PET	Cutinase TfCut2, LC-Cutinase, carboxyl esterase TfCa (Plasmid: pET-20b(+))	*Thermobifida fusca* KW3	[60]
*E. coli* XL1-Blue	PET	PETase (Plasmid: pET32a)	*Ideonella sakaiensis* 201-F6	[61]
*E. coli* BL21 DE3	PET	LC-cutinase (Plasmid: PET28; PJ912)	Plant compost metagenome	[62]
*E. coli* Rosetta-gami B	PET	PETase (Plasmid: pET15b; pET15a)	*Ideonella sakaiensis*	[63]
*E. coli* BL21-CodonPlus (DE3) RIPL	PET	PETase (Plasmid: pET-21b)	*Ideonella sakaiensis*	[64]
*E. coli* C41 (DE3)	PET	PETase (Plasmid: pET-21b(+))	*Ideonella sakaiensis* 201-F6	[65]
*E. coli* BL21 (DE3)	PET	PETase (Plasmid: pET28a)	*Ideonella sakaiensis*	[66]
*E. coli* Rosetta-gami B	PET	Multiple modified *Is*PETase (*Is*PETaseS121E/D186H/R280A)	*Ideonella sakaiensis*	[67]
*E. coli* DH5α	PET	LC cutinase (Plasmid: pHK-LCC)	Plant compost metagenome	[68]
*E. coli* BL21 (DE3)	PET	LC cutinase (Plasmid: pHK-LCC)	Plant compost metagenome	[68]
*Clostridium thermocellum* DSM1313	PET	LC cutinase (Plasmid: pHK-LCC)	Plant compost metagenome	[68]
*E. coli* BL21 (DE3)	PET	Hydrolases 1 and 2 (BTA1 and BTA2); cutinase (FsC); *Is*PETase; leaf-branch compost cutinase (LCC)	*Thermobifida* *fusca* (BTA1 and BTA2); *Fusarium solani pisi* (FsC); *Ideonella sakaiensis* 201-F6 (*Is*PETase); leaf compost metagenome (LCC)	[69]
*E. coli* MG1655 RARE	PET	Terephthalate 1,2-dioxygenase, dihydroxy-3,5-cyclohexadiene-1,4-dicarboxylic acid dehydrogenase, carboxylic acid reductase, catechol O-methyltransferase	*Ideonella sakaiensis*	[70]
*E. coli* PHL628	PET	PETase bound to BIND platform	*Ideonella sakaiensis*	[71]
*E. coli* TOP10	PET	PETase bound to BIND platform	*Ideonella sakaiensis*	[71]
*E. coli* BL21 (DE3)	PET	Leaf-branch compost, cutinase (LCC) and variants	Leaf compost metagenome	[72]
*E. coli* DH5α	PET	Leaf-branch compost, cutinase (LCC) and variants	Leaf compost metagenome	[72]
*E. coli* NEB5α	PET	*Is*PETase; *Is*PETase-MHETase chimera	*Ideonella sakaiensis*	[73]
*Vibrio natriegens*	PET	*Is*PETase; *Is*PETase-MHETase chimera	*Ideonella sakaiensis*	[73]
